# Impact of Nutrition on Academic Performance of First-cycle Primary School Children in Amhara Region, Ethiopia: A Multicenter Cross-Sectional Study

**DOI:** 10.4314/ejhs.v35i5.4

**Published:** 2025-09

**Authors:** Ermiyas Endewunet Melaku, Aklile Semu Tefera

**Affiliations:** 1 Department of Internal Medicine, Debre Berhan University, Debre Berhan, Ethiopia; 2 Department of Epidemiology, Debre Berhan University, Debre Berhan, Ethiopia

**Keywords:** Nutrition, Academic Performance, students, Primary school, Education

## Abstract

**Background:**

Primary school age is a critical period marked by rapid physical growth and significant mental development. While brain development is largely guided by genetic factors, it is also influenced by environmental elements such as nutrition. This study aimed to evaluate the impact of nutritional status on the academic performance of first-cycle primary school children in the North Shoa Zone, Amhara Region, Ethiopia.

**Methods:**

A cross-sectional study was conducted from January 21 to August 30, 2023, using a simple random sampling technique. Data were collected through face-to-face interviews using a structured questionnaire, as well as document reviews. Data entry was performed using Epi Data, and analysis was carried out using STATA version 14. Results were summarized using frequency tables and graphs. Binary logistic regression was used to identify variables associated with academic performance, and significant variables (p < 0.05) were further analyzed using multivariable logistic regression.

**Results:**

A total of 514 students participated in the study. Approximately 56% demonstrated good academic performance. Factors significantly associated with academic performance included maternal education level (AOR = 4.08, 95% CI: 1.60–10.40), regular breakfast consumption (AOR = 4.25, 95% CI: 2.16–8.37), body mass index (AOR = 4.12, 95% CI: 3.04–5.57), and dietary diversity score (AOR = 3.33, 95% CI: 1.99–5.57).

**Conclusions:**

The study revealed a relatively low level of academic performance among students in the study area. Maternal education, regular breakfast intake, healthy BMI, and higher dietary diversity were identified as key factors positively associated with academic achievement. Interventions aimed at improving child nutrition and parental education could enhance academic outcomes in this population.

## Introduction

Child undernutrition remains a major global health challenge, particularly in low- and middle-income countries. Nutritional status is influenced by dietary intake, the presence of disease, general health, and healthcare practices—all of which indirectly affect educational performance. Health has been widely recognized as a key determinant of academic achievement (1). Investing in child nutrition is essential for human capital development, as it is fundamental to children's physical growth, cognitive development, school performance, and long-term productivity. Well-nourished children are better prepared to learn (2).

To achieve their academic potential, children require a diet rich in essential nutrients such as vitamins, minerals, proteins, and fats. However, many children do not receive adequate nutrients from their current diets. Studies have shown that students who maintain better diet quality—characterized by higher intake of fruits and vegetables and lower intake of total fat—exhibit improved academic outcomes (3). Primary school age represents a crucial phase of both rapid physical growth and mental development. While brain development follows a genetic trajectory, it is significantly influenced by environmental factors, including nutrition (4). Other factors, such as parental education, socioeconomic status, access to healthcare, and life experiences, also play a crucial role in shaping brain development (5).

Undernutrition negatively affects academic performance, intellectual development, and school attendance among children and adolescents. Conversely, good nutrition has been linked to improved cognitive and behavioral development, as well as higher school attendance rates (6, 7). Several studies from different countries have consistently shown a strong association between undernutrition and poor academic performance in school children (8–11). Specifically, poor academic achievement has been linked to factors such as skipping breakfast, parental education levels, larger family size, micronutrient deficiencies (e.g., vitamin B12, folic acid, iron), and low dietary diversity (12–14).

In Ethiopia, there is limited research exploring the relationship between nutrition and academic performance among students. To address this gap, the present study aims to assess the impact of nutritional factors on the academic performance of first-cycle primary school children in the North Shoa Zone, Amhara Region, Ethiopia. By investigating these associations, this study seeks to provide valuable insights to guide interventions aimed at improving both the nutritional status and academic success of Ethiopian primary school children. The findings have the potential to inform evidence-based strategies that address nutrition-related challenges and contribute to better educational outcomes for young learners

## Methods and Materials

**Study setting**: A cross-sectional study was conducted between January 21 and August 30, 2023, across four randomly selected primary schools located in different woredas (districts) of North Shoa Zone, Ethiopia. The schools included were Keyit Primary School, Shola Gebeya Primary School, Deneba Primary School, and Tarma Ber Primary School. Students were randomly selected from each school based on proportional representation relative to the total number of students in each grade and school.

### Study Population and Variables

**Study population**: First-cycle primary school students (grades 1–4) enrolled in the selected schools during the study period.

**Study variables**: Academic performance of first-cycle primary school students is the dependent variable while socio-demographic factors (age, sex, parental education and occupation, family income, religion, family size, place of residence, grade level) are the independent variables.

**Dietary factors**: Breakfast consumption, daily meal frequency, 24-hour dietary diversity score (DDS), body mass index (BMI).

**Sample size and sampling procedure**: The sample size was calculated using a single population proportion formula with the assumptions of a 95% confidence interval, 5% margin of error, and an estimated prevalence of 34%. Based on these parameters, a total of 539 students were selected using a systematic random sampling technique (15).

**Data collection instruments and procedures**: Data were collected through face-to-face interviews using a pretested structured questionnaire administered by trained data collectors. Information was gathered on socio-demographic and dietary factors. A focused clinical examination was conducted to assess nutritional status.

**Weight measurement**: A digital weighing scale (Seca 787) with a 250 kg capacity was used to record weight.

**Height measurement**: A non-distensible measuring tape (200 cm) was used to measure height in the Frankfort position, ensuring standardized posture.

**Dietary diversity score**: A 24-hour dietary recall checklist covering 12 food groups, based on the Food and Agriculture Organization (FAO) guidelines, was used to calculate dietary diversity scores (DDS) (16). DDS was categorized as low (≤4), medium (5–8), and high (9–12).

**Academic performance**: Students' academic records were reviewed to obtain their previous semester results.

**Data analysis**: Data were coded, cleaned, and checked for completeness before entry into EpiData version 3.1. Statistical analyses were performed using STATA version 14. Descriptive statistics, including means, standard deviations, frequencies, and percentages, were calculated. Logistic regression analysis was employed to identify factors associated with academic performance. Variables with a p-value <0.2 in bivariate analysis were included in the multivariable logistic regression model to control for potential confounders. Associations were considered statistically significant at p < 0.05. Adjusted odds ratios (AOR) with 95% confidence intervals (CI) were reported.

The following definitions of terms are used.

**Good academic performance**: Semester average score ≥ 50 in the previous term.

**Poor academic performance**: Semester average score < 50 in the previous term.

**Minimum Dietary Diversity Score (DDS)**: Consumption of at least four of the following seven food groups in the previous day:

⚬ Grains, roots, and tubers
⚬ Legumes and nuts
⚬ Dairy products
⚬ Flesh foods
⚬ Eggs
⚬ Vitamin A–rich fruits and vegetables
⚬ Other fruits and vegetables

**Ethical considerations**: All precautions according to the Declaration of Helsinki (Finland, June 1964) were taken to protect the privacy and confidentiality of the personal information of those involved in the research. Ethical clearance and approval to conduct the research was obtained from Institutional Review Board (IRB) office. Official letter was written from the IRB office to each primary school prior to conducting the research. Informed written consent was obtained from the guardians or parents, who were properly informed of the objectives, methods, and institutional affiliations of the researchers and assured that their responses would be kept anonymous and confidential. Respondents (both guardians and students) were clearly told that they had the right to refuse or terminate at any point of the interview or procedure. Moreover, assent was also obtained from children. The interview occurred during a Day-Off for students.

## Results

**Socio-demographic characteristics of students and parents**: A total of 514 students participated in the study, yielding a response rate of 95.36%. Of these, 55% were male, with a mean age of 10.5 ± 0.28 years. The majority (81.7%) lived with both parents, and 54% were from urban areas. Most students (63%) were enrolled in grades 3 and 4. A large proportion of mothers (79.3%) were married. Regarding education, approximately 75% of fathers and 67% of mothers had completed at least primary education. The average family size was 5.67 ± 0.07 (Table 1).

**Table 1:** Sociodemographic characteristics of the study participants at selected first-cycle primary schools of North Shoa Zone, January 21-Augest 30/2023(N=514)

Variable and Category	Frequency	Percent
Sex		
Male	285	55.45
Female	229	44.55
Residence		
Urban	276	53.7
Rural	238	46.3
Child living with		
Both Father and mother	420	81.7
Mother only	52	10.12
Father only	28	5.45
other	14	2.73
Mother's education		
Cannot read and write	78	15.17
Read and write only	109	21.20
primary school completed	174	33.85
Secondary completed	78	15.17
College and above	75	14.59
Father's Education		
Cannot read and write	45	8.75
Read and write only	91	17.70
Primary school completed	186	36.12
Secondary completed	85	16.54
College and above	107	20.82
Mother's Occupation		
Farmer	231	46.02
Government employee	182	36.25
Merchant	62	12.35
Others	27	5.42
Father's Occupation		
Farmer	211	44.14
Government employee	145	30.33
Merchant	86	17.99
Others	36	7.53
Religion of parents the child living with		
Orthodox	447	87.05
Muslim	44	8.5
Protestant	15	3.0
other	8	1.5
Income		
<2500	251	49.70
2500-5000	189	37.43
5000 and above	74	12.87

**24-Hour individual dietary diversity status of the students**: When assessing the individual dietary diversity within the 24 hours preceding the survey, all participants reported consuming starchy staples. More than 90% of the children had consumed legumes, nuts, or seeds during this period. Approximately 43% and 44.36% of the students reported consuming fruits and vegetables, respectively.

In contrast, a significant proportion of students lacked animal-source foods in their diets: 70.62% did not consume meat or meat products, and 58.95% did not consume milk or milk products in the past 24 hours. Additionally, around 55% of the children did not use iodized salt in their meals.

**Figure 1:**
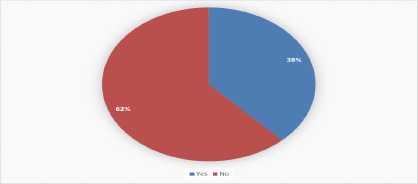
24-hour Minimum dietary diversity score (DDS) of the students at selected first-cycle primary schools of North Sha Zone, from January 21-Augest 30/2023(N=514)

Only 39.49% (203 children) reported eating breakfast before going to school. Furthermore, just 41.44% (213 children) had three or more meals per day (Table 2, Figure 1).

**Table 2:** Food groups consumed by students at selected first-cycle primary schools of North Shoa Zone, January 21-Augest 30/2023(N=514)

Variables	Category	Frequency	Percent
Staple Starches	Yes	514	100
	No	0	0
Legumes/nuts and seeds	Yes	468	91.05
	No	46	8.95
Other Vegetables	Yes	228	44.36
	No	286	55.64
Other Fruits	Yes	221	43.08
	No	292	56.92
Vitamin A rich fruits and vegetables	Yes	96	18.68
	No	418	81.32
Meat and Meat Products	Yes	151	29.38
	No	363	70.62
Milk and milk products	Yes	211	41.05
	No	303	58.95
Egg	Yes	175	34.05
	No	339	65.95

**Figure 2:**
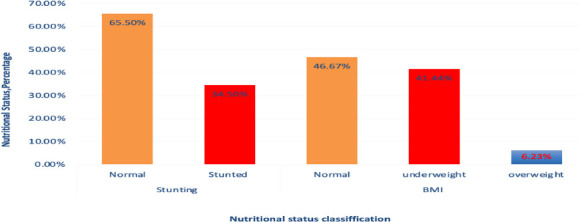
Nutritional status of the students at selected first-cycle primary schools of North Shoa Zone, January 21-Augest 30/2023(N=514)

**Nutritional assessment**: The prevalence of stunting and underweight among students were 34.5 % and 41.43 %, respectively. About 52 % of stunted students and 56.4 % of underweight students were females (Figure 2).

**Academic performance of students**: Out of 514 students, 287 (56 %) of the students scored good academic performance on their previous semester average results. When selected individual subjects were considered, 63.03 %, 53.5 %, 55.64 %, and 49.34 % of students scored good academic performance in Amharic, English, Science, and Mathematics respectively (Table 3).

**Table 3:** Previous Semester Academic performance of children at selected primary schools of North Shoa zone, January 21-Augest 30/2023(N=514)

Variables		Frequency	Percentage
Amharic score	Good	324	63.03
	Poor	190	36.97
English score	Good	275	53.50
	Poor	239	46.5
Science	Good	286	55.64
	Poor	228	44.36
Mathematics	Good	254	49.34
	Poor	260	50.66
Semester average	Good	287	55.84
	Poor	227	44.16

**Factors associated with academic performance of students**: The potential risk variables were entered into the bivariate analysis (binary logistic regression) to check for possible association with the dependent variable (academic performance of students). Accordingly, mothers' education level, mothers' occupation, sex of the children, age of the children, residency of the children, family size, eating breakfast, dietary diversity score, and BMI were found to have p values of less than 0.2. Variables with p values less than 0.2 in the binary logistic regression were entered into the multivariate logistic regression.

After computing the multiple logistic regression, mothers' education level, eating breakfast, dietary diversity score, and BMI of students were found to have a statistically significant association with students' academic performance at P value of less than 0.05 (Table 4).

**Table 4:** Binary and multiple logistic regression analysis of predicted variables of academic performance of children at selected primary schools of North Shoa Zone, January 21-Augest 3 0/2023 (N=514)

Variables	Category	Academic performance		Crude OR with 95% CI	Adjusted OR with 95%) CI	P value

		Good	Poor			
Mothers Education	read and write only	54	51	2.7647	4.083529	0.003*
				(1.422-5.373)	(1.603-10.398)	
	Primary education	54	51	4.1989	4.720083	0.0001*
	completed			(2.268-7.773)	(2.032-10.960)	
	secondary			5.117777	3.000349	0.047*
	education completed	54	51	(2.475-10.578)	(1.015-8.869)	
	Higher Education	54	51	4.141762	1.728435	0.299
	completed			(2.026-8.465)	(0.615-4.849)	
	cannot read and write	18	47			
				………		
Mothers' occupation	Government employer	134	75	1.7866	1.183057	0.761
				(1.226-2.604)	(0.400-3.495)	
	Merchant	24	20	1.2	1.065628	0.911
				(0.630-2.283)	(0.3483-3.259)	
	Farmer	124	124	----------		
Family size	……..	……	…..	0.8635 (0.772-0.964)	1.142775 (0.955-1.367)	0.145
Residency	Urban	146	92	1.5194	0.5609965	0.271
	Rural	141	135	(1.068-2.160)	(0.2003-1.570)	
age of children	………	…	…	0.8849807 (0.776-1.008)	0.9548829 (0.872-1.046)	0.321
Sex of children	Male	170	115	1.415087	1.1296	0.656
	Female	117	112	(0.996-2.009)	(0.660-1.932)	
Breakfast	Yes	175	28	11.10491	4.253101	0.0001*
	No	112	199	(7.0016-17.6)	(2.160-8.371)	
Minimum	Yes	157	38	6.00668	3.332915	0.0001*
Dietary	No	130	189	(3.95-9.131)	(1.994-5.568)	
Diversity Score						
	Normal	194	75	6.445464	4.115393	0.0001*
BMI of children	Low	61	152	(3.588-6.029)	(3.0414-.568)	

## Discussion

This study was aimed to assess the determinants of the academic performance of children at selected first cycle primary schools of North Shoa zone. Accordingly, about 56 % of students were found to have good academic performance in their previous semester results. Studies conducted in USA, Asia, and other African countries showed better student school performance compared to this study (17-20). The possible explanations for this discrepancy could be variations in socio-economic status including income levels, parental education, and access to educational resources and school settings including quality of infrastructure, teaching methods, and available learning resources across different regions.

Compared to similar study conducted in Ethiopia, Nekemte (32.2 %), this study revealed better academic performance of primary school students at the study area(21). The discrepancy in the findings could be the time gap between the two studies, the multi-center nature of this study, and the geographic variation of the study areas.

Maternal educational status was found to have a significant association with good academic performance in which children of mothers who can read and write and completed primary and secondary school education were found to have better academic performance compared to those children who had mothers who cannot read and write. Those children who had mothers who could read and write had 4 times better academic performance compared to those children whose mothers could not read and write (AOR=4.083529, CI: 1.603-10.398).

More specifically, children with mothers who can read and write exhibited a fourfold improvement in academic performance (AOR=4.083529, CI: 1.603-10.398), while those with mothers who completed primary education showed a threefold better academic performance compared to those children in which their mother cannot read write (AOR=3.000349, 95 % CI: 1.015-8.869). Additionally, children of mothers who completed secondary school education displayed approximately 4.7 times better academic performance (AOR=4.720083, CI: 2.032-10.960) compared to those children with mothers who ca did not read and write.

The positive relationship between maternal education and good academic performance of students can be explained by the fact that educated mothers are often better equipped to provide educational support to their children, serve as positive role models for their children, mothers with higher levels of education are better able to communicate effectively with teachers, understand educational expectations, and advocate for their children's needs within the school system and are more likely to encourage their children to aspire to higher levels of education. Other studies conducted in China, Pakistan, Kenya, Ethiopia, and systematic reviews also showed that maternal educational level was positively associated with good academic performance(22-26).

Students who ate their breakfast were found to have about 4.2 times good academic performance compared to those students who did not take their breakfast (AOR=4.253101, 95 % CI:2.160-8.371). Improved academic performance is associated with eating breakfast, in which eating breakfast improves concentration and alertness, increases memory and cognitive function, helps stabilize mood, and contributes to a positive emotional state, creating a more conducive environment for learning and social interaction in the academic setting. Similar studies conducted in China, Canada, and USA also revealed a positive association between eating breakfast and academic performance of school children.(27-29)

A statistically significant correlation was observed between dietary diversity score and academic performance. Students who had access to minimum dietary diversity demonstrated academic performance approximately three times better than students who did not have access for minimum dietary diversity (AOR=3.332915, 95 % CI: 1.994-5.568). This positive correlation could be explained by the fact that A diverse diet is more likely to provide a range of essential nutrients needed for optimal cognitive function and may positively influence cognitive abilities and academic performance. A balanced and varied diet also helps maintain an adequate supply of energy to the brain. This can impact attention, focus, and overall cognitive performance. Similar studies conducted in Canada, Morocco and Nigeria also showed similar associations between DDS and academic performance of primary school children (30-33)

Students who had a normal BMI showed approximately four times higher academic performance compared to those students with underweight BMI (AOR=4.115393, CI=3.0414-5.568). The observed positive association between academic performance and BMI could be explained by the possibility that individuals with normal BMIs may have improved access to nutrition, potentially leading to positive effects on brain function and academic achievement. Other comparable research studies conducted in USA, Pakistan, Nigeria, and Ethiopia have also indicated a positive correlation between BMI and academic performance (18,34-36).

In conclusion, the findings of this study revealed a notable low academic performance of first cycle primary school students in the study area. Maternal education, breakfast intake, BMI, and vitamin B12 levels were identified as associated factors with academic performance. Encouraging and facilitating higher levels of maternal education can have a positive impact on the academic performance of students.

Educational initiatives aimed at mothers can contribute to creating a supportive learning environment at home. Efforts should be made to ensure that students have access to nutritious meals. School-based nutrition programs and community initiatives can play a crucial role in improving overall dietary habits. Regular monitoring of vitamin B12 levels among students is essential. Addressing any deficiencies through dietary interventions or supplements can positively influence cognitive function and, subsequently, academic performance. In conclusion, a holistic approach that combines education, nutrition, and community engagement is vital for improving the academic performance of primary school students in the study area.
